# Parent-Metabolite Pharmacokinetic Modeling of Formononetin and Its Active Metabolites in Rats after Oral Administration of Formononetin Formulations

**DOI:** 10.3390/pharmaceutics15010045

**Published:** 2022-12-23

**Authors:** Ju Hee Kim, Dong Wook Kang, Seok-jin Cho, Hea-Young Cho

**Affiliations:** College of Pharmacy, CHA University, 335 Pangyo-ro, Bundang-gu, Seongnam-si 13488, Republic of Korea

**Keywords:** UPLC-MS/MS, formononetin, daidzein, dihydrodaidzein, equol, active metabolites, pharmacokinetics, parent-metabolite pharamcokientic modeling, plasma protein binding

## Abstract

Formononetin is a major isoflavone contained in propolis and is reported to exhibit various pharmacological effects. However, the use of formononetin in pharmaceutical industry is limited due to its low bioavailability and solubility. There had been several efforts on formononetin formulation development, but further study is required to acquire optimal formulation. The aim of this study is to conduct pharmacokinetic (PK) evaluations after the oral administration of three formononetin formulations (20 mg/kg) in male Sprague Dawley rats. Then, a parent-metabolite PK model for formononetin was developed and evaluated for the first time. To do this, a simultaneous analysis method for formononetin and its active metabolites, daidzein, dihydrodaidzein and equol in rat plasma was developed using ultra-performance liquid chromatography tandem mass spectrometry. The separation was performed using a gradient elution of water and acetonitrile and a Kinetex C_18_ column (2.1 mm × 100 mm, 1.7 µm particle size) at a temperature of 30 ± 5 °C. The simultaneous analytical method developed in this study was validated according to international guidance and was successfully applied for the pharmacokinetic study. The time-plasma concentrations of formononetin and daidzein were well described by a two-compartment model combined with a metabolite compartment. Additionally, plasma protein binding assay was conducted in male rat plasma. The findings from the study could be used as a fundamental for the future development of formononetin as a pharmaceutical product.

## 1. Introduction

Propolis, also known as bee glue, refers to a combination of beeswax and saliva produced by bees for constructing and maintaining beehives. It is known to have effects in treating inflammation, bacterial or fungal infection, acute ulcer, and cancer. Among several flavonoids and phenolic acid contained in propolis, an isoflavone called formononetin (FMN) is identified as the major constituent [1]. FMN itself is known to have several pharmacological activities including. but not limited to, anti-cancer; cellular neuroprotection; insulin resistance and hyperglycemia reduction as well as kidney damage attenuation in type 2 diabetes [2,3,4,5,6]. However, due to FMN’s low bioavailability and water solubility [7], its pharmaceutical use has been limited so far. There were several attempts to overcome the physico-chemical limitation by formulation development including poly (lactic-co-glycolic acid)-nanoparticle and hydroxypropyl-β-cyclodextrin complex [8,9,10]. However, further studies on FMN formulation is required to enhance its bioavailability sufficiently. Co-crystallization and solid dispersion technology are two representative methods known to enhance solubility and bioavailability [11,12]. Therefore, the aim of this study was to conduct pharmacokinetic evaluation of FMN formulations: solution, co-crystallization, and solid dispersion. 

**Figure 1:**
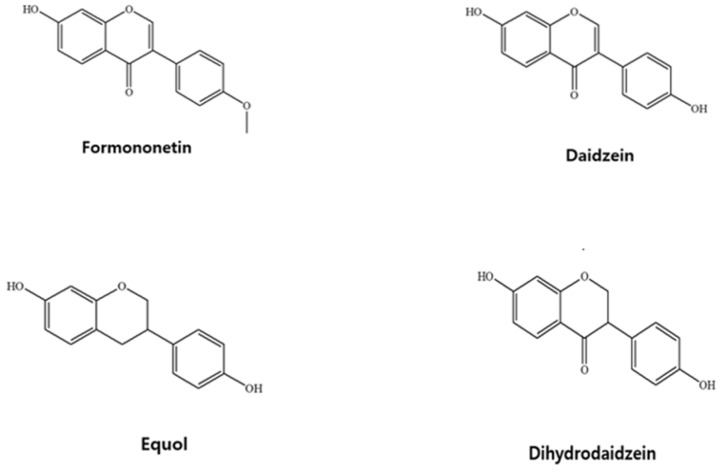
The chemical structures of FMN, DZN, EQL, and DHD.

FMN is metabolized into bioactive compounds known as daidzein (DZN) by gut microflora and cytochrome P450 isoforms 1A2, 2C9*1, 2A6, and 2C19 [13]. Then, DZN is further converted into two other bioactive compounds dihydrodaidzein (DHD) and equol (EQL) [14]. Thus, all four bioactive compounds including FMN, DZN, DHD and EQL were selected as target analytes in this study. The chemical structures of four target analytes of this study is depicted in Figure 1. 

To the best of our knowledge, several analytical methods were reported to analyze FMN and/or other metabolites using HPLC-DAD [15], LC-MS/MS [16,17], and UPLC-MS/MS [18,19]. Among these, only one publication reported simultaneous analytical methods of FMN, DZN, DHD and EQL. Prasain et al. [16] have developed a rapid two-minute LC-MS/MS method that allowed quantification of 11 phytoestrogen metabolites, including the four isoflavone of our interest, in human plasma. However, the method required using 200 μL of human plasma sample. This method cannot be adapted in rat experiment since only 50–100 μL rat plasma per time point can be collected. Therefore, a simultaneous UPLC-MS/MS method for the more sensitive and rapid determination of FMN, DZN, DHD and EQL using minimal plasma was developed for the pharmacokinetic evaluation.

Several studies have been conducted on FMN pharmacokinetics in rats after oral administration of plant extracts or decoction [18,20,21]. However, there are only few studies where FMN itself was administered for pharmacokinetic evaluations [15,22]. When 2–50 mg/kg FMN was orally administered, the peak plasma concentration reached between 0.5–1 h and the maximum plasma concentration (C_max_) ranged between 17 ng/mL and 81 ng/mL. The clearance of FMN was estimated to be 5 L/h/kg for intravenous administration. There is no report on pharmacokinetic modeling or parent-metabolite modeling of FMN so far.

In this study, pharmacokinetic evaluation was conducted after the oral administration of FMN solution and FMN formulations in male Sprague Dawley rats using the novel analysis method. Then, a pharmacokinetic model for FMN and its metabolite after oral administration of FMN was developed and validated for the first time. Additionally, protein binding assay for all four compounds were conducted. 

## 2. Materials and Methods

### 2.1. Chemicals and Reagents

FMN, DZN, DHD, EQL and DZN-d4 were purchased from Sigma-Aldrich (St. Louis, MO, USA). Methanol and acetonitrile were purchased by J.T. Baker (Phillipsburg, NJ, USA). Formic acid and Acetic acid were purchased from Sigma-Aldrich (St Louis, MO, USA). Evoqua Water Technologies (Pittsburgh, PA, USA) was used to purify water. All chemicals had the highest grade or quality available. Male Sprague Dawley rat plasma was purchased from BioChemed Services (Winchester, United Kingdom). The FMN formulations were prepared by Pharmaceutical Formulation Design Laboratory in College of Pharmacy, CHA University. FMN was mixed with polyvinylpyrrolidone K30 and D-α-Tocopherol polyethylene glycol 1000 succinate then dried in a vacuum oven to prepare amorphous solid dispersion. FMN cocrystal was prepared by grinding formononetin with imidazole. FMN, FMN solid dispersion and FMN cocrystal were each suspended in 1% HPMC solution just before the oral administration to rats.

### 2.2. Animal Study Design

Sprague Dawley rats were chosen as the experiment animal for the study since it was reported that identical metabolites were converted from FMN from rat liver microsome reaction and human liver microsomes [13]. 15 male Sprague Dawley rats (221.70 ± 4.90 g) were purchased from Dae Han Bio Link Co., Ltd. (Chungchungbuk-do, Republic of Korea) for the animal experiment. The experiment was conducted in accordance with the Guidelines for the Care and Use of Laboratory Animals and was approved by the Institutional Animal Care and Use Committee (IACUC200084). The animals were maintained in the temperature and humidity range of 25 ± 1 °C and 50 ± 5% RH, respectively, over the acclimation period. To minimize the effects of food on pharmacokinetic profile of isoflavones, the animals were fasted overnight with free access to water. 

Animals were allocated equally into 3 groups (5 rats per group). FMN solution dissolved in 1% hydroxypropyl methylcellulose (HPMC), FMN co-crystal and FMN solid dispersion were orally administered at a dose of 20 mg/kg to group 1, 2, and 3, respectively. Approximately 200 μL of blood samples were collected in heparinized Eppendorf tubes from the jugular vein before the administration and 0.08, 0.16, 0.33, 0.5 1, 2, 4, 8, 12, and 24 h after the oral administration. The blood samples were immediately centrifuged (10,000× *g*, 10 min, 4 °C) to obtain plasma samples and was stored at −80 °C.

### 2.3. Analytical Methodology

Determination of the four isoflavones and DZN-d4 (internal standard, IS) in samples were conducted using Acquity UPLC system (Waters Corp., Milford, MA, USA) coupled to Xevo TQ-S triple quadrupole mass spectrometry (Waters Corp., USA) with electrospray (ESI) source. The chromatographic separations of the four isoflavones was performed in a Kinetex C18 column (2.1 × 100 mm, 1.7 μm particle size, Phenomenex, Torrance, CA, USA) at 40 ± 0.5 °C. The mobile phase consisted of water (mobile phase A) and acetonitrile (mobile phase B) with gradient elution at a flow rate of 0.2 mL/min. The composition of the mobile phase changed as follows: 0–1.0 min (70% B), 1.0–1.5 min (30% B), 1.5–4.0 min (30% B), 4.0–4.1 min (70% B), 4.1–5.0 min (70% B). The mass spectrometer was operated using an electrospray ionization (ESI) interface in negative ion mode with multiple reaction monitoring (MRM) transitions, such as m/z 267.16 → 252.18, 253.14 → 132.20, 255.19 → 149.12, 241.14 → 121.17, and 257.15 → 136.21 for FMN, DZN, DHD, EQL and the IS, respectively. The optimized collision energy of FMN, DZN, DHD, EQL and IS were −22, −38, −22, −15 and −38, respectively. Data acquisition and analysis were achieved using Masslynx 4.1 software (Waters Corp., USA).

Individual standard stock solutions of FMN, DZN, DHD, EQL and the IS were prepared by dissolving accurately weighed standard compounds in methanol at a concentration of 1 mg/mL and were stored at −20 °C. The standard working solutions of four isoflavones (2, 5, 10, 50, 100, and 200 ng/mL) and the IS (10 μg/mL) were diluted with 50% methanol in water from the standard stock solutions. The samples for the standard calibration curves were prepared by spiking 5 μL of the standard working solutions in 45 μL of blank rat plasma. The quality control (QC) samples of four levels with 2, 6, 80, and 160 ng/mL were prepared for the evaluation of accuracy and precision. The samples for the calibration and QC were freshly prepared on the day of analysis.

The samples were extracted by protein precipitation using methanol. 50 μL of rat samples were added with 10 μL of the IS solution (10 μg/mL of DZN-d4 in 50% methanol) to correct the loss of analytes during sample preparation. 100 μL of methanol was added to the mixed sample, vortexed for 1 min and centrifuged at 15,000× *g* for 5 min. Then, 5 μL of aliquots were injected into the UPLC-MS/MS system. The method validation was performed according to the FDA guideline for industry bioanalytical method validation.

### 2.4. Plasma Protein Binding Assay

The plasma protein binding of FMN, DZN, DHD and EQL was estimated using Ultra-filtration method using Centrifree^®^ micro-partition system (Amicon Inc., Lexington, MA, USA). The experiment was conducted at concentrations of 50, 150, 500 and 1000 ng/mL (n = 3) for all four compounds in male SD rat plasma. The spiked plasma samples were allowed to equilibrate for at least 15 min before it was put into Centrifree^®^ ultrafiltration tubes. The tubes were centrifuged at 1000× *g* for 15 min at 37 °C. Then, compound concentrations in the ultrafiltrate buffer (*C_UF_*) and plasma in the sample reservoir (*C_total_*) were quantitated by the anlytical method described in Section 2.3. The plasma protein binding rate was calculated using the following formula:

$$Plasma\;protein\;binding\;\left(\%\right)=\frac{C_{total}-C_{UF}}{C_{total}}\times 100$$

### 2.5. Pharmacokinetic Evazluation and Model Development

PK parameters such as the maximum plasma concentration (C_max_) and the time to reach C_max_ (T_max_) were determined from plasma concentration-time curve. The linear trapezoidal rule was used to calculate area under the curve to the final measured concentration (AUC_0-t_), then area from C_last_ to area expolated to infinity was added to AUC_0-t_ to integrate AUC_0-∞_. The half-life (t_1/2_) was calculated as 0.693/k and the volume of distribution (Vd/F) as dose/(k × AUC_0-∞_). The PK analysis was performed using WinNonlin^®^ software (version 8.2, Certara™ Company, Princeton, NJ, USA). Then, a PK model was developed for FMN and its metabolites following oral administration of solution or formulations. The model was implemented in the WinNonlin model with NLME engine and estimated using the naïve-pooled and First Order Conditional Estimation-Extended Least Squares (FOCE-ELS) algorithms. The random effects of PK parameters were exponentially modeled for the description of inter-individual variability (IIV) as follows:

$$P_{i}=P_{TV}\times \exp\left(\eta_{i}\right),$$

where *P_i_* is the estimated PK parameter for ith individual, *P_TV_* is the typical value of the PK parameter, and *η_i_* is a random variable for the ith individual following a normal distribution with the mean of zero and a variance of ω^2^. The intra-individual variability (ε) was described by proportional, additive, or log-additive error model. 

One- and two-compartment models with first-order kinetics with or without lag time were tested to describe the absorption and disposition of FMN and the metabolites. Optimal model selection was based on diagnostic values such as twice the negative log like twice the negative log like (−2LL), Akaike information Criterion (AIC), Bayesian Information Criteria (BIC), as well as visual inspection of various diagnostic plots (goodness of fit plot) and precision of parameter estimates. For non-parametric evaluation of the model bootstrap analysis was conducted.

In the modeling process, the decrease of −2LL by more than 3.84 according to an additional parameter or the increase of −2LL by more than 6.63 according to an eliminated parameter was considered a significant improvement of the nested model. The goodness-of-fit plots and bootstrap was conducted for the evaluation of the developed model. In this study, goodness-of-fit plots such as the conditional weighted residuals versus predictions and the dependent variable versus the individual predictions were used for both parents and metabolite model. The bootstrap generated 1000 replicates datasets from the orginal data sets to repeatedely fit the model for median values and 95% confidence intervals of parameters. 

### 2.6. Statistical Analysis

Statistical significance was evaluated through Kruskal–Wallis test using software (IBM SPSS Statistics for Windows, Version 29.0, IBM Corp., Armonk, NY, USA) with *p* < 0.05 inferring significant difference.

## 3. Results and Discussion

### 3.1. Analytical Method Validation

**Figure 2:**
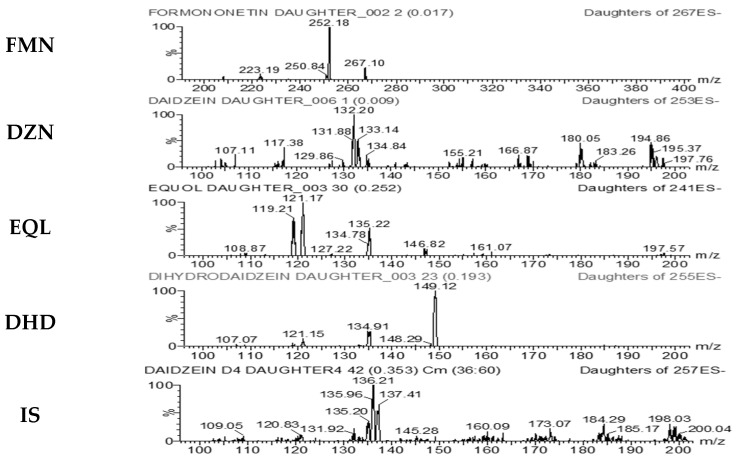
The product ion scan spectra with chemical structures of FMN, DZN, EQL, DHD, and IS.

**Figure 3:**
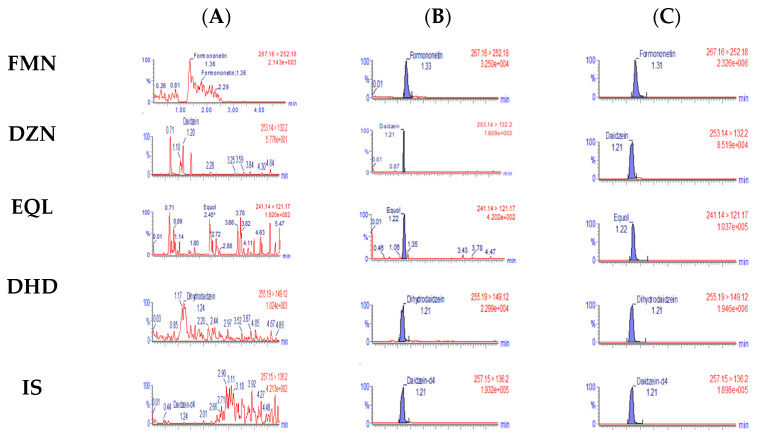
Representative multiple reaction monitoring chromatogram of (**A**) blank plasma sample (**B**) blank plasma spiked with target analytes and IS at LLOQ concentration and (**C**) blank plasma spiked with target analytes and IS at ULOQ concentration.

The product ion scan spectra of FMN, DZN, EQL, DHD and IS are presented in Figure 2. The representative chromatograms of blank sample, blank sample spiked with target analytes and IS (LLOQ or ULOQ concentration) are presented in Figure 3. 

The calibration curves for the four isoflavones in rat plasma showed a linearity over the concentration range of 2–200 ng/mL with a correlation coefficient (r^2^) in the range of 0.996–0.998. The linear regression equations of the calibration curves with plotting the peak area ratio (y) of analytes to the IS versus the nominal concentration (x) of analytes with weighted (1/x^2^) were: y = (0.053 ± 0.007) x + (0.051 ± 0.005) for FMN, y = (0.0023 ± 0.0001) x + (0.0003 ± 0.0007) for DZN, y = (0.056 ± 0.002) x + (0.005 ± 0.008) for DHD, and y = (0.0022 ± 0.0004) x + (0.0001 ± 0.0019) for EQL. 

Table 1 summarizes the intra- and inter-batch precision and accuracy evaluation of the four isoflavones at four concentration levels: LLOQ (2 ng/mL) and QC samples at 6, 80, and 160 ng/mL in rat plasma. The intra-batch accuracy ranged from 90.06 to 100.90% for FMN, 97.83 to 106.33% for DZN, 97.03 to 99.17% for DHD, and 92.29 to 103.50% for EQL with a precision (%CV) of <13.43% for FMN, <13.03% for DZN, <6.13% for DHD, and <14.94% for EQL, respectively. The inter-batch accuracy ranged from 98.91 to 104.17% for FMN, 93.33 to 104.88% for DZN, 97.72 to 101.10% for DHD, and 95.61 to 106.33% for EQL with a precision (%CV) of <8.72% for FMN, <9.34% for DZN, <5.85% for DHD, and <6.31% for EQL, respectively. 

The results were within the acceptable criteria of ±15% for QC samples and ±20% for LLOQ demonstrating a satisfactory precision, accuracy, and reproducibility.

### 3.2. Plasma Protein Binding Assay Result

The plasma protein binding assay was conducted at four different concentration levels in male rat plasma samples for all four compounds. The result is summarized in Table 2. All compounds had relatively high plasma protein binding rate in concentration range of 50–1000 ng/mL, and there was no statistically significant difference in plasma protein binding ratio between the concentrations in all four compounds. 

Protein binding assay were conducted to study the basic characteristics of the four analytes, since there is no previous report on protein binding rate for FMN (in range of 500–1000 ng/mL) or DZN, EQL, and DHD in the range of 50–1000 ng/mL. The protein binding assay results of FMN was similar to the previous reports which were 93.61 ± 0.44% and 96.14 ± 0.15% at 50 ng/mL and 150 ng/mL [15] FMN in female rat plasma, respectively. For DZN, plasma protein binding assay was reported in human serum albumin. Song et al. [23] have determined unbound DZN at concentration levels of 3–15 μM DZN in which the unbinding drug was 0.889–6.398 μM, respectively. 

### 3.3. Pharmacokinetic Evaluation of FMN and Its Metabolites

**Figure 4:**
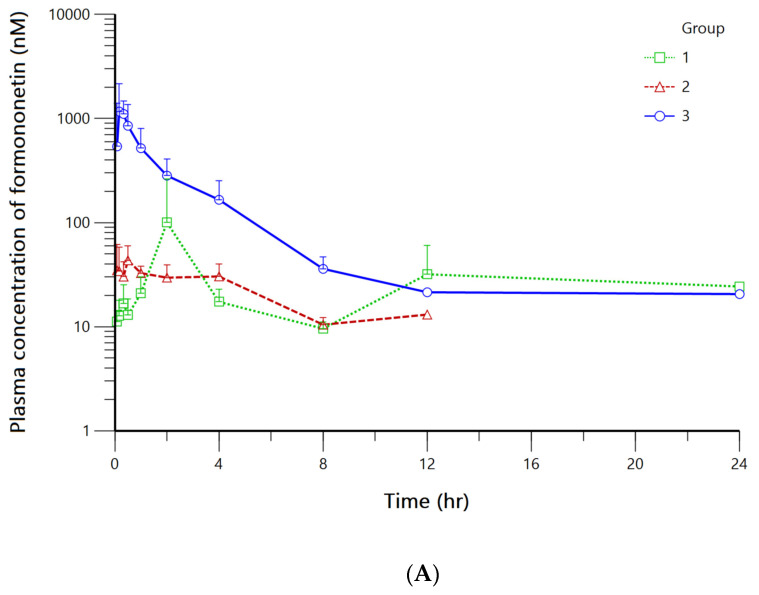

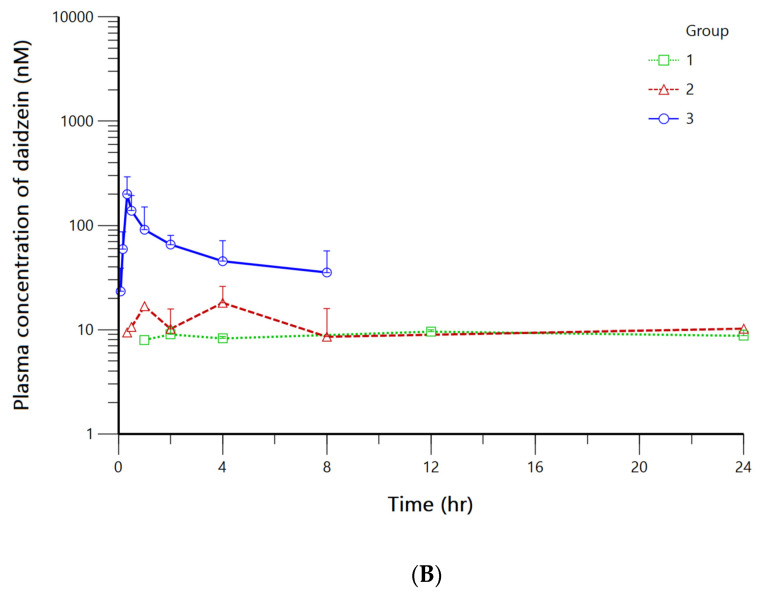
The plasma concentration of (**A**) FMN and (**B**) DZN after oral administration of 20 mg/kg FMN solution (Group 1, □), 20 mg/kg FMN co-crystal (Group 2, △), and 20 mg/kg FMN solid dispersion (Group 3, ○) in Sprague Dawley rats (The error bars in the graph represent standard deviations).

The validated simultaneous UPLC-MS/MS method was applied to measure plasma concentrations of FMN and its metabolites after oral administration of FMN solution and formulations in 15 male SD rats. The bioanalysis results are depicted in Figure 4, and the estimated pharmacokinetic parameters for all three groups using non-compartmental analysis is summarized in Table 3 and Table 4.

In all three groups, only FMN and DZN was detected after the oral administration of FMN. Thus, pharmacokinetic evaluation and parent-metabolite modeling for FMN and DZN was conducted. Since the time versus mean plasma FMN concentration profiles of group 1 and group 2 failed to clearly manifest disposition or absorption pattern, respectively. Thus, the temporal change data of FMN plasma concentration for group 3 was used for the pharmacokinetic modeling. 

### 3.4. Parent-Metabolite PK Model Devleopment

**Figure 5:**
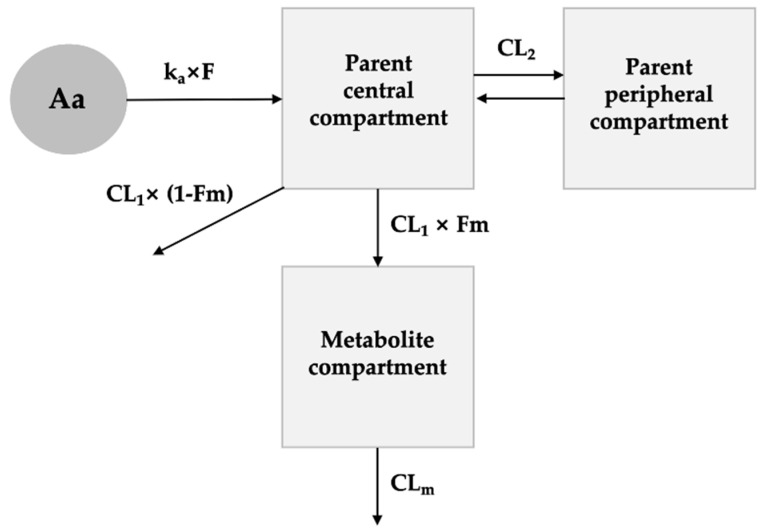
Parent-metabolite PK model scheme for FMN and DZN following the oral administration of FMN solid dispersion. (CL: clearance of FMN; CL_m_, clearance of DZN, k_a_: absorption rate constant; F, fraction of FMN absorbed; Fm, fraction of FMN metabolized).

The obtained non-compartmental analysis data for group 3 was used as initial parameters for the compartmental modeling. One- and two-compartment models with first-order absorption, and with or without lag time were tested to fit the FMN profile following the oral administration. The clearance was tested as first-order and non-linear Michaelis-Menten type. After the selection of the optimal structural model for the parent compound, metabolite compartment was added to complete a parent-metabolite pharmacokinetic model. Two-compartment model with first-order absorption without lag time was identified as the optimal model that best described the plasma profiles of FMN. The DZN was best fitted to metabolite-compartment with first-order elimination. The final PK model is depicted in Figure 5, and the diagnostic values obtained from the model trials are summarized in Table 5.

The final model selected was two-compartment parent model with a metabolite compartment. The structural models were considered based on the diagnostic values including −2LL, AIC and BIC. As AIC and BIC are useful in comparing structural models [24], it was used to favor model M1 over M3. Although the AIC of M3 was smaller than M1, M1 was still favored since a drop in AIC(or BIC) of 2 is considered a threshold for selecting a better model [24]. When selecting error model, parameter CV (%) was considered. 

**Figure 6:**
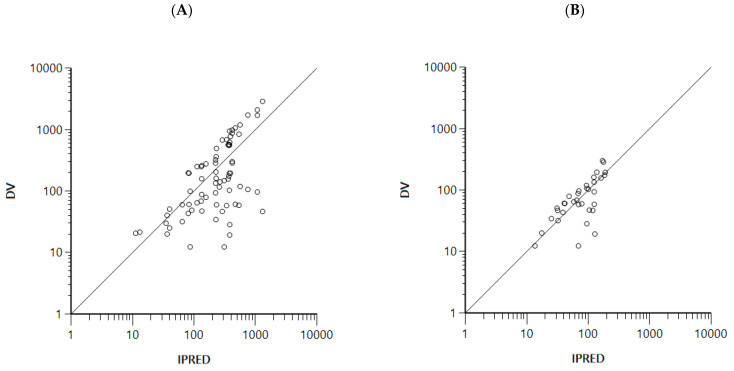

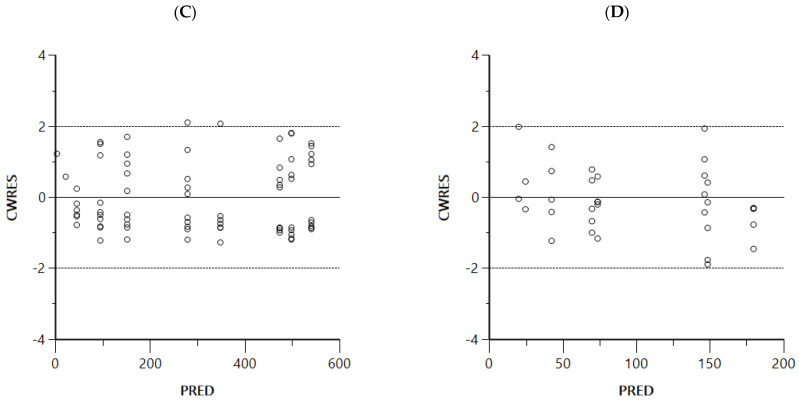
Goodness-of-fit plots for the final model. Observations vs. individual predicted concentrations of (**A**) FMN and (**B**) DZN, and conditional weighted residuals (CWRES) vs. population predictions of (**C**) FMN and (**D**) DZN. Circles represent the observed values of FMN and DZN.

**Figure 7:**
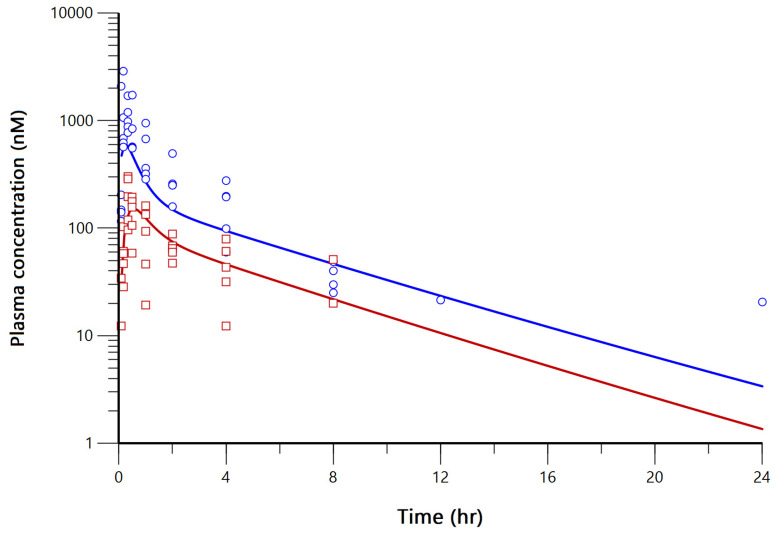
Observed and predicted plasma concentration-time plot of FMN (observed: ○, predicted: straight blue line) and DZN (observed: □, predicted: straight red line) of FMN solid dispersion oral administration group.

Evaluation of the final model was conducted using goodness-of-fit plots and visual predictive check. As shown in Figure 6, no significant bias was captured in the model. The model adequately explained the general trend of the data. In addition to this, bootstrapping was conducted for the evaluation of robustness and predictive performance of the optimal model. The 95% interval results produced by boostrapping are provided in Table 6, and all parameter estimations were well within the 95% interval. The estimated PK parameters are listed in Table 6. The model-predicted plasma concentration-time plots of FMN and DZN fitted to the observed data of group 3 are shown in Figure 7. 

In all groups, only FMN and DZN was detected after the oral administration of FMN. This results concurs with a previous in vitro study result [13] that reported DZN as the major metabolite produced after incubation of FMN in human liver microsomes. Furthermore, our result agrees with the previous report by Raju et al. [25]. Raju et al. have administered 5 mg/kg FMN intravenously or 10 mg/kg FMN orally to SD rats, then they conducted the bioanalysis of plasma samples for quantitation of FMN, DZN and EQL. However, they were able to quantitate only FMN and DZN in rat plasma. In our study, the dose has doubled (20 mg/kg FMN), but still EQL as well as DHD was not quantitated. 

According to a previous study [22], the C_max_ and AUC_inf_ of 302.1 ± 35.9 nM and 757.7 ± 48.2 nM × hr was reported after the oral administration of 20 mg/kg FMN dissolved in 0.5% CMC-Na to male Sprague Dawley rats. In our study, the C_max_ and AUC_inf_ was 110.03 ± 158.20 nM and 368.12 ± 166.13 ng × hr/mL after the administration of 20 mg/kg FMN dissolved in 1% HPMC. The difference between the parameters can be explained by the solvents in which FMN was dissolved in. In our study, 1% HPMC was used for FMN solution to keep the variable minimal between the three groups. In other study [15], plasma concentration of FMN and DZN were quantified after oral administration of FMN to female SD rats at 50 mg/kg dose in 0.25% CMC suspension. 

Although the C_max_ and AUC_inf_ of group 2 did not significantly differ from group 1, it can be seen that Group 3 had major enhancement in the parameters since the C_max_ and AUC_inf_ increased by approximately 12-fold and 3-fold, respectively, compared to group 1. Thus, the solubility and bioavailability of FMN was greatly improved when orally administered in solid dispersion formulation. 

The optimal model was two-compartment model with first-order absorption and linear elimination for parent and one-compartment model with linear elimination for metabolite. As mentioned in Introduction, FMN and DZN is reported to go through gut microflora metabolism. However, as gut microflora metabolism of the analyte in rat or the significance of gut metabolism is not reported so far, it was not considered in the current modeling. 

The oral bioavailability was parameterized as “F” for estimation through the developed model. Various studies used compartmental pharmacokinetic modeling or physiologicall-based pharmacokinetic modeling approaches on estimation of F when the drug information or data is limited in the early drug development phases. 

The oral bioavailability(F) of FMN solution dissolved in 0.5% CMC-Na in rats was reported to be 21.8% [22]. The estimated fraction of FMN dose absorbed (F) was 0.31, so the solid dispersion formulation could have increased the bioavailability of FMN; however, as the solvent in which FMN was dissolved in is different, direct comparison is not possible with the reported bioavailability of 21.8%. The fraction of CL_1_ into metabolite compartment was estimated to be 0.89. The information on FMN metabolism in rat is limited, a study reported that about 25% of FMN was excreted intact in humans [26].

## 4. Conclusions

PK evaluation of three FMN formulations were conducted using a rapid and sensitive simultaneous bioanalysis method developed and validated according to bioanalysis guidelines of FDA. FMN solid dispersion showed the highest C_max_ and AUC_inf_. Furthermore, a parent-metabolite PK model for FMN and its bioactive compound was developed for the first time. In addition to this, plasma protein binding assay was conducted for FMN, DZN, DHD, and EQL. This study will provide fundamentals for further development of FMN as a pharmaceutical product. 

## Figures and Tables

**Table 1:** The precision and Accuracy of the determination of FMN, DZN, DHD and EQL in rat plasma.

**FMN**			
Theoretical concentration (ng/mL)	Mean measured concentration (n = 5, ng/mL)	Precision (%CV)	Accuracy (%)
**Inter-day**			
2	2.06	2.12	103.00
6	5.93	7.76	98.91
80	81.87	4.21	102.34
160	166.67	8.72	104.17
**Intra-day**			
2	2.01	13.43	100.90
6	5.40	6.16	90.06
80	78.41	13.19	98.01
160	158.98	0.67	99.36
**DZN**			
Theoretical concentration (ng/mL)	Mean measured concentration (n = 5, ng/mL)	Precision (%CV)	Accuracy (%)
**Inter-day**			
2	1.93	9.34	96.61
6	5.60	6.72	93.33
80	83.91	2.61	104.88
160	164.02	1.10	102.51
**Intra-day**			
2	2.13	9.19	106.33
6	5.87	13.03	97.83
80	84.99	5.78	106.24
160	165.27	4.17	103.29
**DHD**			
Theoretical concentration (ng/mL)	Mean measured concentration (n = 5, ng/mL)	Precision (%CV)	Accuracy (%)
**Inter-day**			
2	1.95	1.28	97.72
6	5.93	5.85	98.83
80	80.88	2.65	101.10
160	156.42	0.68	97.76
**Intra-day**			
2	1.98	4.55	99.17
6	5.55	3.37	92.50
80	78.46	6.13	98.08
160	155.25	3.70	97.03
**EQL**			
Theoretical concentration (ng/mL)	Mean measured concentration (n = 5, ng/mL)	Precision (%CV)	Accuracy (%)
**Inter-day**			
2	2.13	6.31	106.33
6	6.09	1.41	101.54
80	76.49	3.50	95.61
160	156.33	2.25	97.71
**Intra-day**			
2	2.07	7.21	103.50
6	6.11	13.76	101.78
80	73.83	14.94	92.29
160	156.55	10.36	97.85

**Table 2:** Plasma protein binding assay result of FMN, DZN, EQL, and DHD.

Compound	50 ng/mL	150 ng/mL	500 ng/mL	1000 ng/mL
FMN	88.44 ± 2.29	93.36 ± 4.85	85.51 ± 1.71	91.80 ± 1.31
DZN	95.44 ± 1.60	96.58 ± 1.82	94.31 ± 0.37	94.69 ± 0.53
EQL	-	98.69 ± 0.53	98.62 ± 0.41	97.80 ± 1.29
DHD	88.15 ± 0.53	87.39 ± 1.31	88.02 ± 0.90	86.83 ± 1.50

**Table 3:** The estimated pharmacokinetic parameters of FMN.

Group	ID	Half-Life	T_max_	C_max_	AUC_last_	AUC_inf_	V_d_/F	CL/F
		(hr)	(hr)	(nmol/L)	(hr × nmol/L)	(hr × nmol/L)	(L)	(L/hr)
1	1	-	2.00	31.39	303.60	-	-	-
	2	-	2.00	390.77	613.56	-	-	-
	3	-	2.00	23.71	184.49	-	-	-
	4	-	12.00	73.96	291.14	-	-	-
	5	-	2.00	30.31	447.80	-	-	-
	Mean	-	4.00	110.03	368.12	-	-	-
	SD	-	4.47	158.20	166.13	-	-	-
2	6	3.89	0.17	48.09	225.00	294.69	325.45	57.99
	7	5.96	0.08	62.63	237.73	316.02	455.13	52.96
	8	-	4.00	46.15	253.12	-	-	-
	9	4.83	0.08	62.67	163.43	236.90	471.02	67.56
	10	3.93	0.50	72.17	201.05	266.28	336.36	59.30
	Mean	4.65	0.97	58.34	216.07	278.47	396.99	59.45
	SD	0.97	1.70	10.98	35.07	34.40	76.72	6.06
3	11	4.34	0.33	1195.61	3563.43	3648.51	28.84	4.60
	12	2.14	0.33	775.35	1741.27	1873.23	27.08	8.78
	13	1.81	0.17	2886.70	2638.59	2701.55	16.21	6.21
	14	2.01	0.33	876.75	1549.70	1644.38	28.92	9.95
	15	2.67	0.33	980.87	1404.79	1470.06	44.20	11.47
	Mean	2.60	0.30	1343.06	2179.56	2267.55	29.05	8.20
	SD	1.03	0.08	876.85	910.34	904.88	9.98	2.79

**Table 4:** The estimated pharmacokinetic parameters of DZN.

Group	ID	Half Life	T_max_	C_max_	AUC_last_	AUC_inf_
		(hr)	(hr)	(nmol/L)	(hr × nmol/L)	(hr × nmol/L)
1	1	*-*	2.00	10.11	102.75	-
	2	-	-	-	-	-
	3	-	12.00	9.87	194.29	-
	4	-	-	-	-	-
	5	-	24.00	9.52	199.56	-
	Mean	-	12.67	9.83	165.53	-
	SD	-	11.02	0.30	54.44	-
2	6	-	4.00	27.10	154.04	-
	7	-	4.00	13.21	31.46	-
	8	-	4.00	14.00	274.84	-
	9	-	-	-	-	-
	10	-	2.00	4.51	28.06	-
	Mean	-	3.50	14.71	122.10	-
	SD	-	1.00	9.31	117.48	-
3	11	-	0.33	118.89	231.05	-
	12	3.40	0.33	195.43	488.82	585.86
	13	-	0.50	106.11	303.33	-
	14	4.60	0.33	301.39	376.39	786.20
	15	1.36	0.33	286.20	358.81	415.60
	Mean	3.12	0.37	201.61	351.68	595.89
	SD	1.64	0.08	90.98	95.34	185.50

**Table 5:** The parent-metabolite PK model development process for FMN and DZN after oral administration of 20 mg/kg FMN solid dispersion.

Model	Description	−2LL	AIC	BIC	No. of Parameters
**Structural model (FMN)**					
P1	One-compartment model without lag time	603.86	613.86	622.43	5
P2	One-compartment model with lag time	603.86	615.86	626.14	6
P3 *	Two-compartment model without lag time	558.62	572.62	584.62	7
P4	Two-compartment model with lag time	612.74	628.74	642.45	8
P5	Two-compartment model without lag time with nonlinear elimination	558.62	576.62	592.04	9
**Structural model (FMN + DZN)**					
M1 *	Two-compartment model with metabolite compartment	1394.32	1416.32	1445.93	11
M2	Two-compartment model with metabolite compartment and nonlinear elimination	1394.32	1420.32	1455.31	13
M3	Two-compartment model with two-compartment metabolite	1388.51	1414.51	1449.50	13
M4	Two-compartment model with two-compartment metabolite and nonlinear elimination	1388.51	1418.51	1458.88	15
**IIV model**					
M1-1	IIV on k_a_, F, V_1_, V_2_, CL_1_, CL_2_, V_m_, CL_m_, F_m_	1418.79	1458.79	1512.62	20
M1-2	IIV on k_a_, F, V_1_, V_2_, CL_1_, CL_2_, V_m_, F_m_	1375.43	1413.43	1464.57	19
M1-3	IIV on k_a_, F, V_1_, CL_1_, CL_2_, V_m_, F_m_	1379.96	1415.96	1464.41	18
M1-4 *	IIV on k_a_, F, V_1_, CL_1_, CL_2_, V_m_	1358.82	1392.82	1438.57	17
M1-5	IIV on F, V_1_, CL_1_, CL_2_, V_m_	1359.07	1391.07	1434.13	16
M1-6	IIV on F, V_1_, CL_1_, V_m_	1359.24	1389.24	1429.81	15
M1-7	IIV on F, V_1_, V_m_	1360.70	1388.70	1426.37	14
M1-8	IIV on V_1_, V_m_	1369.55	1395.55	1430.54	13
M1-9	IIV on V_1_	1379.75	1403.75	1436.04	12
**Error model**					
M1-4-1	Proportional	1358.82	1392.82	1438.57	17
M1-4-2	Additive	1992.30	2026.30	2072.05	17
M1-4-3 ^†^	Log-additive	1358.82	1392.82	1438.57	17
M1-4-4	Additive + proportional	1358.87	1396.87	1448.01	19

* Selected model. ^†^ final model.

**Table 6:** The model estimated pharmacokinetic parameters for FMN and DZN.

Parameters (Unit)	Description	Estimate	CV (%)	Bootstrap Results	
				2.5% CI	97.5% CI
**Fixed effect**					
F	Fraction of FMN dose absorbed	0.31	20.34	0.19	0.45
Fm	Fraction of CL_1_ into metabolite compartment	0.89	10.56	0.61	0.99
k_a_ (1/hr)	First-order absorption rate constant	7.12	50.47	2.74	20.79
V_1_ (L)	Volume of distribution of central compartment	25.04	15.01	16.62	36.41
V_2_ (L)	Volume of distribution of peripheral compartment	48.31	40.19	27.08	88.12
V_m_ (L)	Volume of distribution of metabolite compartment	10.46	41.77	4.29	33.04
CL_1_ (L/hr)	Total clearance of FMN	19.85	15.35	12.62	26.14
CL_2_ (L/hr)	Inter-compartmental clearance of FMN	38.68	53.50	9.43	80.12
CL_m_ (L/hr)	Total clearance of DZN	41.54	29.24	21.53	62.86
**Random effects**					
ω_V1_	IIV of V_1_	0.18	42.43	0.00	0.45
ω_CL1_	IIV of CL_1_	0.05	22.36	0.00	0.17
ω_ka_	IIV of k_a_	0.25	50.00	0.00	0.38
ω_CL2_	IIV of CL_2_	0.11	33.17	0.00	0.33
ω_F_	IIV of F	0.03	17.32	0.00	0.08
ω_Vm_	IIV of Vm	1.20	109.54	0.00	2.54
**Residual error**					
ε_1_	Proportional error of FMN	0.75	3.53	0.63	0.89
ε_2_	Proportional error of DZN	0.42	21.55	0.27	0.63

## Data Availability

The data presented in this study are available on request from the corresponding author. The data are not publicly available due to privacy issues.

## Abbreviations

FMN	Formononetin
DZN	Daidzein
DHD	Dihydrodaidzein
EQL	Equol
IS	Internal Standard
QC	Quality control
PK	pharmacokinetics
SD	Standard deviation
